# A High-pressure Solution for a High-pressure Situation: Management of Cerebral Air Embolism with Hyperbaric Oxygen Therapy

**DOI:** 10.7759/cureus.5559

**Published:** 2019-09-03

**Authors:** Robert P Murphy, Julie Donnellan

**Affiliations:** 1 Internal Medicine, University Hospital Galway, Galway, IRL; 2 Anaesthesiology, University Hospital Galway, Galway, IRL

**Keywords:** air embolism, stroke, hyperbaric oxygen therapy

## Abstract

Cerebral air embolism can complicate many medical procedures, including cardiac surgery, venous and arterial access, and laparoscopic surgery. It can be a devastating diagnosis and can cause a life-threatening compromise to the cardiac, respiratory, or cerebrovascular system. It is a rare complication of central venous vascular access manipulation. A cerebral air embolism can lead to acute ischemic and cerebral oedema, which mimics other stroke syndromes, but the acute treatment differs, with prompt administration of hyperbaric oxygen therapy being the mainstay of treatment.

A 59-year-old male became acutely unresponsive followed by the emergence of evolving neurology with fixed gaze palsy and a dense 0/5 left-sided hemiparesis. This occurred shortly after a right internal jugular central venous catheter (CVC) was removed (against protocol) during inspiration and sitting upright. Computed tomography (CT) imaging showed air in the right internal jugular vein, as well as intraparenchymal air. Treatment with hyperbaric oxygen was instituted within six hours. There was an excellent recovery of neurologic function, with power improving to 4+/5 over the course of the following week.

Clinical staff need to be aware of the policy for central line removal, as well as having a high index of suspicion for air embolism in patients with evolving neurology immediately post-line removal. Early consideration of hyperbaric oxygen can result in improved functional outcomes.

## Introduction

A cerebral air embolism can complicate many medical procedures, including cardiac surgery, venous and arterial access, and laparoscopic surgery. Air embolism can affect the cardiovascular, respiratory, or cerebral systems and cause life-threatening sequelae [1]. It is a potentially fatal complication of central venous access. A cerebral air embolism can cause severe neurological symptoms due to ischaemia and cerebral oedema. The presentation can mimic acute stroke syndromes but the treatment differs. Rapid identification and treatment with hyperbaric oxygen therapy can improve outcomes when instituted early [2].

## Case presentation

A 59-year-old man suffered an out-of-hospital ventricular fibrillation cardiac arrest. He was resuscitated on the scene by emergency personnel and transferred to the hospital. In the emergency department, a right internal jugular central venous catheter (CVC) was placed to aid in resuscitation. An urgent angiogram was performed. Significant stenosis was also identified in the left anterior descending artery and the right coronary artery. There was an acute occlusion of a small marginal branch of the left circumflex artery which was too small for percutaneous intervention. A decision regarding percutaneous revascularisation was deferred until the patients’ neurologic recovery from prolonged out-of-hospital resuscitation became clear. He was commenced on dual antiplatelet therapy.

He made an excellent neurological recovery and was being managed in the coronary care unit (CCU) following a transfer from ICU. In anticipation of his discharge to a ward, his central venous catheter was removed. This was done in an upright position sitting in a chair and during inspiration. Shortly afterward, the patient became acutely unwell; he had chest pain, was diaphoretic, and became unresponsive. His cardiac output was maintained throughout. He was unresponsive for three minutes. As he became more responsive, his evolving neurology became obvious, with right facial droop, left-sided inattention, and profound left-sided weakness with power 0/5 in both upper limb and lower limb. His blood pressure and glucose were both normal.

**Figure 1:**
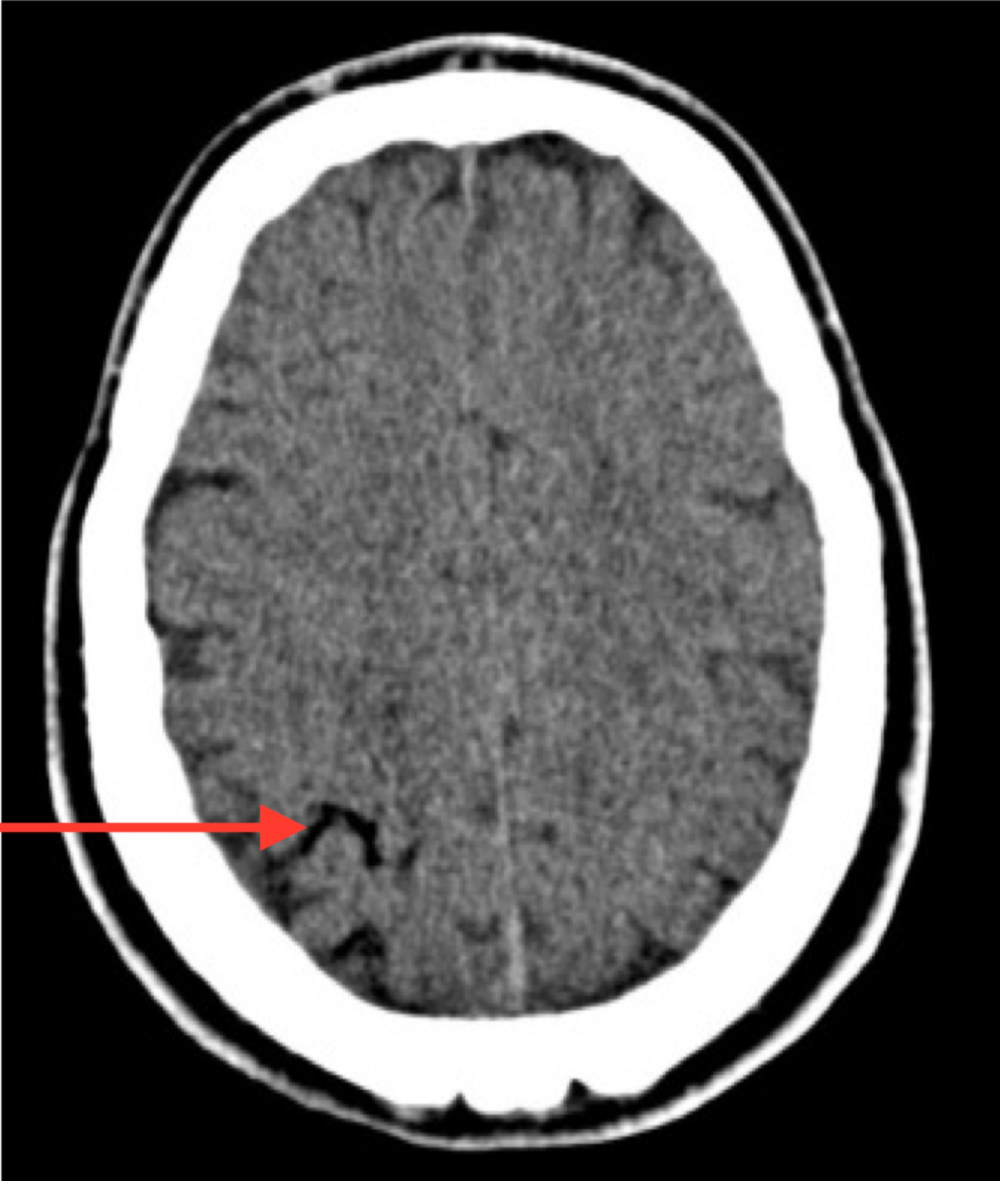
Coronal slice of computed tomography (CT) of the brain with a red arrow demonstrating intraparenchymal air

Computed tomography (CT) of the brain and a CT angiogram were carried out. The brain CT showed an area of acute hypo-attenuation within the posterior aspect of the right frontal lobe consistent with air within the cortical vein (Figure 1).

**Figure 2:**
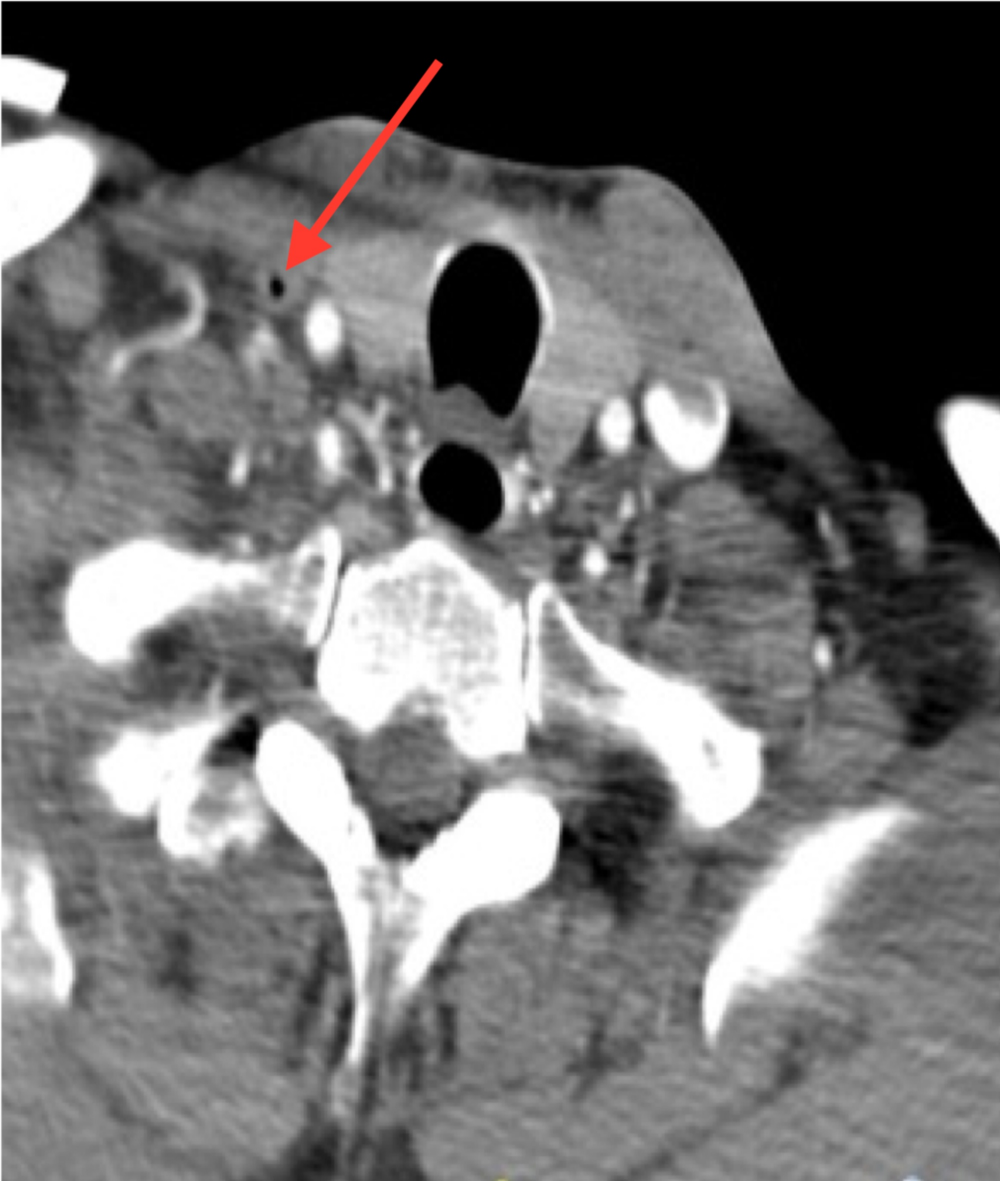
Coronal slice from the computed tomography (CT) angiogram at the level of the trachea with a red arrow showing air in the right internal jugular vein

There was also evidence of air within the right internal jugular vein (Figure 2). Subsequent magnetic resonance (MRI) of the brain confirmed an acute infarct. 

Transoesophageal echocardiogram did not reveal any evidence of patent foramen ovale (PFO), atrial septal defect, or any evidence of left ventricular (LV) thrombus post-myocardial infarction (MI). The CT angiogram did not demonstrate any evidence of acute thrombus, and there was no evidence of any haemorrhage on the CT of the brain.

**Figure 3:**
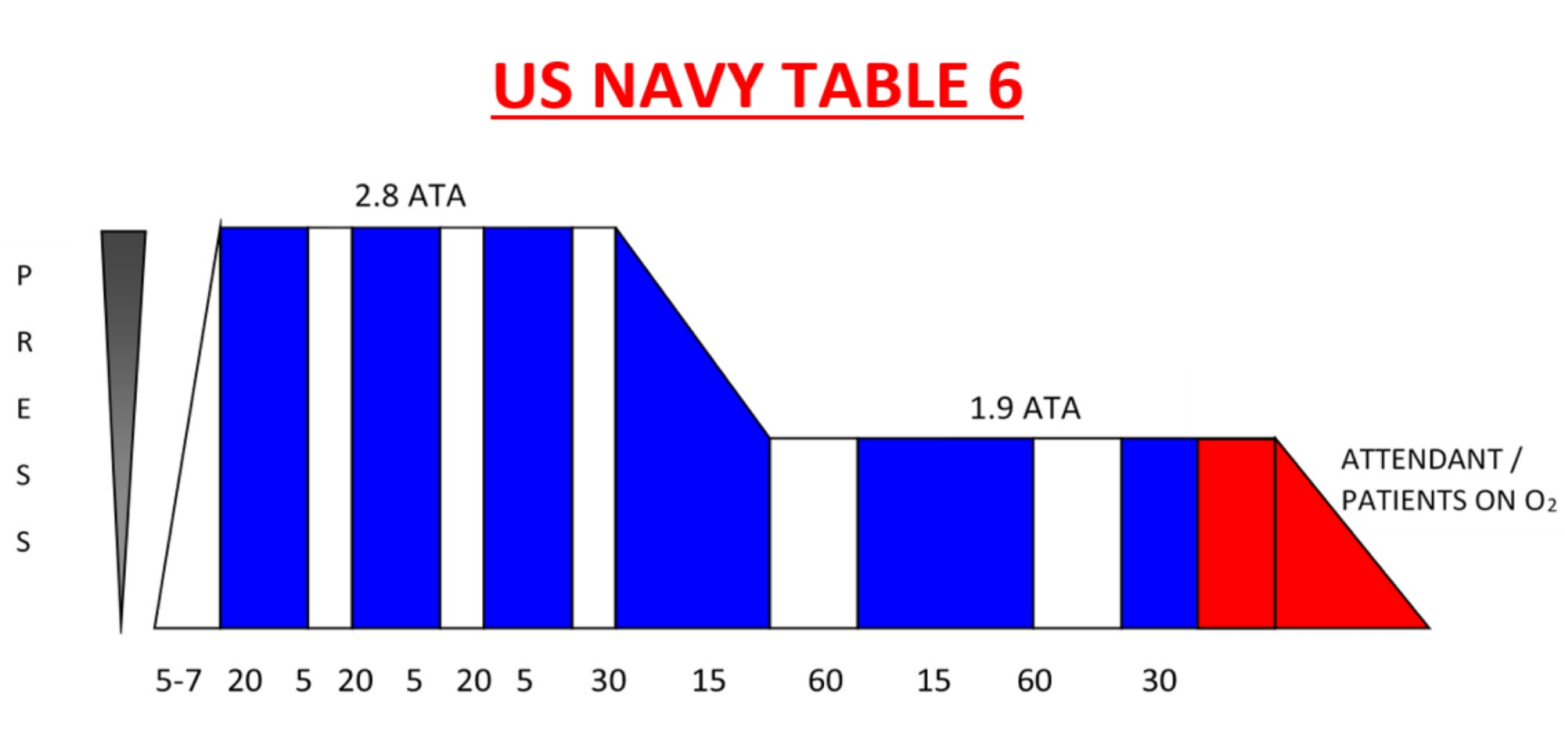
Description of the United States (US) Navy table pressure Oxygen (O_2_) is delivered to the patient during the time periods indicated by the blue bars with air delivered as indicated by the white bars. ATA stands for 'atmospheres absolute' and is a term used to measure the pressure of the air surrounding us at any given time.

On suspicion of an air embolism, the patient was immediately placed in the left lateral decubitus position (Durant’s manoeuvre) with the head tilted down to prevent further air traveling into the cerebral circulation. The Hyperbaric Oxygen Service reviewed the patient and the United States Navy (USN) Table 6 was prescribed (Figure 3, Table 1). Treatment began within six hours of the injury. This involved a hyperbaric team of three personnel and placement of a temporary tympanostomy tube under local anaesthetic by an ear, nose, and throat specialist. An intensive care nurse accompanied the patient during treatment in the hyperbaric chamber. A USN Table 6 provided 100% oxygen at a maximum pressure of 2.8 ATA (atmospheres absolute) with a total treatment time of 4 hours, 45 minutes, not including the compression time. The treatment was well-tolerated without any haemodynamic instability. The patient underwent five hyperbaric treatments in total. Subsequent hyperbaric treatments were of shorter duration.

**Table 1:** Description of the Oxygen/Air Cycles O_2_: oxygen

Total Time	Depth	Gas	Time (Mins)
20	18	O_2_	20
25	18	Air	5
45	18	O_2_	20
50	18	Air	5
1:10	18	O_2_	20
1:15	18	Air	5
1:45	18 to 9	O_2_	30
2:00	9	Air	15
3:00	9	O_2_	60
3:15	9	Air	15
4:15	9	O_2_	60
4:45	9 to 0	O_2_	30

During this time, the patient was managed in the stroke unit where he also received intensive multidisciplinary rehabilitation input. He made an excellent neurological recovery, and power improved to 4+/5.

## Discussion

A cerebral air embolism can be a devastating injury and is often iatrogenic. The estimated incidence of iatrogenic air embolism is 2.65 per 100,000 hospitalizations [3]. It can be a complication of insertion, manipulation, or removal of central catheters, with a reported incidence of 0.03% to 2% [4]. A cerebral air embolism is usually due to arterial injection of air or due to paradoxical air embolism traveling from the venous system to the arterial system via a shunt, e.g., patent foramen ovale. Retrograde flow of air in the venous system is a rare cause of cerebral air embolism [5].

The echocardiogram was normal and thus did not suggest a paradoxical embolism via a septal defect or through a PFO. The CT pulmonary angiogram did not reveal any evidence of pulmonary arteriovenous malformation which, if present, could account for air passing from the venous to the arterial system.

CT imaging showed air within the right internal jugular vein and pneumocephalus consistent with air within the right cortical vein. These neuroradiological findings, along with the lack of a PFO and the lack of cardiopulmonary findings, indicate the likely mechanism was retrograde flow within the venous system [6]. Similar cases have been identified in the literature in keeping with the retrograde mechanism of transmission of the gas [7].

In this case, air bubbles ascended against the venous blood flow to lodge in the cerebral system. Air can rise in an upright patient to the cerebral venous circulation at a greater speed than the venous blood flow due to its low specific gravity [5].

A high index of suspicion is needed for a prompt diagnosis and treatment of a cerebral air embolism. While the need to avoid air embolism during insertion and manipulation of a central venous catheter is appreciated, the risk associated with removal of the catheter is not as widely understood [8]. There is a need for increased physician awareness of the risk, as well as education, about ways in which to correctly position patients to try and minimise this risk [9]. The standard practice which must be followed includes lying the patient flat with the site of the CVC below the level of the heart before removal in order to prevent the entry of air into the vein [10]. Emergency management by all physicians should also include administering 100% oxygen to reduce embolus volume by eliminating nitrogen. 

Any neurologic symptoms in close temporal association to catheter insertion, manipulation, or removal must be investigated. CT may not show the presence of air in the cerebral vasculature, especially if there is a delay in imaging [11]. A CT was done within 30 minutes of the symptom onset in this case and revealed an area of hypo-attenuation in the right frontal lobe. The clinical presentation, coupled with air in the vein and the removal of a CVC, meant that the most likely aetiology for this presentation was venous air embolism. A subsequent MRI scan of the brain confirmed the acute infarct.

The presentation of a cerebral venous air embolism can mimic a thromboembolic stroke with symptoms of focal neurological deficits [12]. Rapid identification and treatment with hyperbaric oxygen therapy can improve outcomes when instituted early [2]. If there is any evidence of a neurological deficit, then hyperbaric oxygen therapy should be considered [13]. With hyperbaric oxygen therapy, the patient breathes 100% oxygen at a pressure above that of atmospheric pressure. Boyle's law states that the size of a gas bubble in a liquid will decrease with increased pressure, and this is the basis for using hyperbaric medicine in the treatment of arterial air emboli. With hyperbaric oxygen, this decreases the size of the gas bubble both by raising the ambient pressure and by causing systemic hyperoxia. Supplemental oxygen also reduces the size of the gas embolus by increasing the gradient for the egress of nitrogen from the bubble.

In this case, a US Navy Table 6 was prescribed, and this is the standard regimen used in such cases. Continued treatment is recommended if there is a continuing clinical response. The main factor determining response is the time from injury to the beginning of hyperbaric treatment. A goal of beginning treatment within six hours has been shown to improve outcomes compared to a more delayed onset [2]. Although early treatment is preferable, there have been reports of good outcomes in patients with delayed onset of treatment [14]. If working in a centre without a hyperbaric oxygen service, it is still worth urgently referring and seeking expert treatment. 

## Conclusions

Cerebral air embolism can cause severe neurological symptoms due to ischaemia and cerebral oedema. Clinical staff need to be aware of the policy for central line removal, as well as having a high index of suspicion for air embolism in patients with evolving neurology immediately following a post-line removal. Early consideration of hyperbaric oxygen can result in improved functional outcomes.
